# Opioid-Induced Hallucinations: A Case Report

**DOI:** 10.7759/cureus.74915

**Published:** 2024-12-01

**Authors:** Arvind Dhanabalan, Sall Saveen, Christina Singh, Ramona Ramasamy, Keerthiga Raveendran

**Affiliations:** 1 Department of Medicine and Surgery, All Saints University School of Medicine, Roseau, DMA; 2 Department of Psychiatry, Louisiana State University Health Sciences Center, Shreveport, USA; 3 Department of Psychiatry and Behavioral Sciences, Louisiana State University Health Sciences Center, Shreveport, USA; 4 Department of Medicine and Surgery, PSG Institute of Medical Sciences and Research, Coimbatore, IND

**Keywords:** drug-induced hallucinations, drug-induced psychosis, opioid-induced hallucinations, postictal hallucinations, schizophrenia

## Abstract

This article presents the case of a 67-year-old African American male patient who was referred to a psychiatry clinic by his Internal Medicine Provider with a diagnosis of "schizophrenia, unspecified." The patient reported the onset of auditory and visual hallucinations (AVHs) two years ago, coinciding with his starting Norco (hydrocodone 5 mg/acetaminophen 325 mg) for chronic back pain. He noted that his AVH worsened when he increased his prescribed Norco dosage (within his prescribed recommended range) and observed that the hallucinations ceased when he discontinued the medication. This case highlights the potential for opioid-induced AVH to be misdiagnosed as schizophrenia while emphasizing the importance of careful evaluation of opioid use in psychiatric assessments.

## Introduction

Schizophrenia is a significant mental disorder that influences an individual's cognition, emotions, and actions. Individuals with schizophrenia may appear to be disconnected from reality, which can be distressing for them and their loved ones. The clinical diagnosis of schizophrenia is made after obtaining a detailed psychiatric history and mental status examination and after ruling out other psychiatric and medical causes of psychosis [1]. One of the common symptoms associated with schizophrenia is hallucinations. A hallucination happens when you receive sensory information that does not exist, a disturbance in perception created by your brain. Various neurotransmitters have been linked to hallucinations, such as the overactivity of dopamine in the subcortical and limbic system [2]. Hallucinations can affect any of your five senses, so you can hear, see, feel, taste, or smell something that no one else can sense. Many people automatically link hallucinations to schizophrenia. Hallucinating alone does not automatically mean you have schizophrenia [3].   There are many other causes for hallucinations, such as side effects of medications, drug withdrawal, delirium, dementia, etc.

One of the less commonly discussed causes of medication-induced hallucinations is due to opioid use. Fentanyl use during postoperative pain management has a reported rate of 6% for hallucinations. The low incidence is likely due to events being unreported because of the tolerable intensity of many hallucinations and fear associated with the stigma of being labeled as psychologically unstable [4]. Despite controversy over its use for non-cancer pain, opioid therapy is used to treat various pain conditions. As the patient-to-physician ratio is expected to increase, opioid therapy is anticipated to be administered by healthcare providers from various specialties. In the United States, opioid prescription for low back pain has increased, and opioids are now the most commonly prescribed drug class. More than half of regular opioid users report back pain. Rates of opioid prescribing in the United States and Canada are two to three times higher than in Europe [5].

This case report presents a 67-year-old African American male patient with a history of lower back pain and drug-induced auditory and visual hallucinations (AVHs).  This case illustrates how opioid-induced AVHs can be mistaken for schizophrenia and emphasizes the need for careful evaluation of opioid use in psychiatric assessments.

## Case presentation

A 67-year-old African American male patient presented with a medical history of congestive heart failure, coronary artery disease, hypertension, peripheral artery disease, persistent atrial fibrillation, gastroesophageal reflux disease, chronic hepatitis C complicated with hepatic coma, chronic midline back pain, and spinal stenosis at L4-L5.  He is also a chronic tobacco smoker with occasional use of marijuana (one to two hits every other month). He was initially referred to the psychiatry clinic via his internal medicine primary care doctor for a diagnosis of schizophrenia, undetermined. The patient had no psychiatric history or symptoms before he was 63; he was diagnosed with schizophrenia after a seizure. The patient also denied any family history of psychiatric illness or dementia. He presented with visual hallucinations along with paranoia, the visual hallucinations were of people trying to attack him and seeing animals that were not present, and the symptoms lasted for 20 days after epileptic seizure. He was admitted to an inpatient mental health facility for treatment, and his symptoms settled with Seroquel 100 mg once a day at bedtime (QHS). After two years of Seroquel use, it was decreased to 50 mg QHS, which was having a beneficial effect on his mood and aiding him in his sleep.

A further plan has been developed to gradually reduce the dosage of Seroquel and to begin taking trazodone. During his last outpatient clinic visit, the patient stated that two years ago, when he was on 50 mg Seroquel, he suddenly developed AVH along with paranoia. It was a sudden onset of symptoms, starting with the feeling of someone following him along with hearing voices that were saying a little of everything. The voices were negative and put him down; they were often just present in the background. However, the voices never told him to harm himself or others. He also had visual hallucinations where he would see little objects on the roof, such as worms running across the house's roof. He was not smoking marijuana or taking street drugs during this time. Due to his worsening back pain, he was increasing his dose of prescribed Norco up to four tablets a day. He noticed a worsening of his AVH when his dosage of Norco increased and stopped the medication himself. The withdrawal of his medication settled his symptoms, and no further episodes of AVH or paranoia have presented since this episode.

## Discussion

This case involves a 67-year-old African American male patient who last experienced AVH two years ago while he was taking Norco for his chronic back pain. Understanding a range of factors that can cause AVH is necessary, and, in our patient, it was important to consider his past psychiatric diagnosis, medications, and any drug use.

When examining the patient's past psychiatric history, we understand he was diagnosed with schizophrenia when he was 63 years old. While one of the common symptoms associated with schizophrenia is hallucinations, that alone does not mean one has schizophrenia [4].  The patient then reported hallucinations and paranoia, but this episode was likely due to postictal psychosis after his seizure. Patients suffer a psychotic outbreak after undergoing a seizure cluster lasting several days, usually after maintaining lucidity for a brief period. The state of mental disturbance that comes on after a seizure may leave the patient with confusion, dementia, hallucinations, delusions, and irritability that leads to violence in rare instances [6].  Furthermore, our patient does not fit the criteria for a primary psychotic disorder like schizophrenia. The Diagnostic and Statistical Manual of Mental Disorders, Fifth Edition, requires that continuous signs of the disturbance persist for at least six months, along with significant social dysfunction as an integral part of the diagnostic criteria for schizophrenia, neither of which fit the description of our patient. Secondary psychosis is more likely to be considered here as there are atypical clinical features, a lack of family history of a primary psychotic disorder, and psychosis occurring at an older age [7].

To rule out the possibility of other medication-induced hallucinations, it is important to explore the patient's regularly consumed medications and their links to hallucinations. We understand that the patient was on baclofen [8], pantoprazole [9], metoprolol [10], gabapentin [11], and trazodone [12] for his various comorbidities. However, none of the above-mentioned medications correlated with his AVH development or cessation in our patient. The patient also disclosed he consumes cannabis recreationally, which can be ruled out as cannabis rarely induces a full-blown psychosis, though transient paranoia is more frequently seen [7]. In a study by Bruera et al., four cancer patients with no previous cognitive impairment developed visual hallucinations on high doses of hydromorphone. The hallucinations resolved after switching to a different opioid and along with the administration of haloperidol [13].  In the case of our patient, his AVH ceased when he stopped taking Norco.

A review of our patient's history shows he was on 50 mg of Seroquel when he developed the AVH. He clearly states that his episode coincided with his increase in Norco dosage and ceased when Norco was stopped. Before this last episode, his last known hallucination was only postictal, making the drug-induced (Norco) hallucination the most likely causality for his episode. Further review of the patient charts shows no documentation about his AVH episode while on Norco, highlighting the importance of careful evaluation of opioid use in patient assessments.

## Conclusions

Pain is a common reason for adults to seek medical care, and opioid medications are frequently used to manage this symptom. Thus, it is imperative that prescribers educate themselves on all the side effects associated with the medication. While there are many well-known side effects of opioid therapy, hallucinations are less commonly discussed. Unfortunately, opioid-induced hallucinations are often underreported or misattributed to underlying or misdiagnosed psychiatric conditions rather than the opioid use itself. All organic causes of hallucinations must be ruled out with thorough investigations before acknowledging a psychiatric component in patients. With the ongoing practice of prescribing opioids, it is essential for physicians to appreciate the importance of thoroughly assessing opioid use during psychiatric evaluations.
